# The Change in Healthcare-Associated Infections in Intensive Care Units Associated with the Coronavirus Disease 2019 in Taiwan

**DOI:** 10.3390/medicina61111971

**Published:** 2025-11-03

**Authors:** Chien-Ying Wang, Yu-Hsuan Chen, Chih-Chun Hsiao, Chun-Gu Cheng, Chun-An Cheng

**Affiliations:** 1 Department of Critical Care Medicine, Taipei Veterans General Hospital, Taipei 112201, Taiwan; 2 Division of Traumatology, Emergency Department, Taipei Veterans General Hospital, Taipei 112201, Taiwan; 3 Faculty of Medicine, School of Medicine, National Yang Ming Chiao Tung University, Taipei 112304, Taiwan; 4 Department of Exercise and Health Sciences, University of Taipei, Taipei 111036, Taiwan; 5 Division of Chest Medicine, Department of Internal Medicine, Cheng Hsin General Hospital, Taipei 11220, Taiwan; 6 Department of Nursing, Taoyuan Armed Forces General Hospital, Taoyuan 32549, Taiwan; 7 Department of Emergency Medicine, Taoyuan Armed Forces General Hospital, Taoyuan 32549, Taiwan; 8 Department of Emergency Medicine, Tri-Service General Hospital, National Defense Medical University, Taipei 11490, Taiwan; 9 Department of Neurology, Tri-Service General Hospital, National Defense Medical University, Taipei 11490, Taiwan

**Keywords:** healthcare-associated infection, intensive care unit, coronavirus disease 2019

## Abstract

*Background and Objectives*: Changes in the incidence of healthcare-associated infections (HAIs) during the coronavirus disease 2019 (COVID-19) pandemic and during periods with fewer or more COVID-19 cases have been inconclusively studied. Compared with 2015, in 2019, the abundances of the microorganisms *Klebsiella pneumoniae* and *Enterococcus faecium* increased in intensive care units (ICUs) in Taiwan. The trend in the incidence of HAIs in ICUs in Taiwan during the emergence of new infectious diseases is worth studying. *Materials and Methods*: We surveyed the incidence densities of different types of HAIs, device-associated HAIs, pathogens, and antimicrobial resistance in a dataset from the Taiwan Healthcare-associated Infection and Antimicrobial Resistance Surveillance System from 2015 to 2022. The change in incidence density trends was evaluated via Poisson regression, and the change in proportion trends was checked via the Mantel–Haenszel chi-square test. *Results*: The incidence of HAIs decreased from 5.7 to 5.17 episodes per 1000 person-days from the pre-COVID-19 period to the post-COVID-19 period. The incidences of healthcare-acquired pneumonia (HAP), device-associated HAIs decreased. However, the incidences of bloodstream infections (BSIs) increased. The percentages of patients with *Escherichia coli*, *Staphylococcus aureus*, *Pseudomonas aeruginosa*, and *Acinetobacter baumannii* infections significantly decreased. The percentage of patients with methicillin-resistant *Staphylococcus aureus* (MRSA) infection decreased, but that of patients with carbapenem-resistant *K. pneumoniae* (CRKP), carbapenem-resistant *Acinetobacter baumannii* (CRAB), carbapenem-resistant *Pseudomonas aeruginosa*, and vancomycin-resistant *Enterococcus faecium* infections increased. The antimicrobial consumption related to CRKP increased and MRSA decreased. *Conclusions*: Overall, HAIs, HAP, and VAP decreased in incidence after the COVID-19 pandemic. These results revealed decreases in MRSA infection incidence under infection control protocols with more antimicrobial use. However, the proportion of CRKP among HAIs increased with broad-spectrum antimicrobial agent use. Based on the recent incidence of HAIs in ICUs, the quality of infection control in medical units can be enhanced to decrease HAI incidence.

## 1. Introduction

Healthcare-associated infections (HAIs) are defined as the acquisition of an infection while receiving care for an illness and being admitted to a healthcare setting for more than 48 h. Device-associated HAIs (DAHAIs) are defined based on occurrence between 1 and 12 months after devices are used [1]. HAIs prolong hospitalization, increase medical spending [2], increase disease severity, and increase mortality [3]. Patients who are susceptible to opportunistic infections and cross-contamination with devices often experience HAIs [4]. Intensive care unit (ICU) mortality associated with HAIs has a positive odds ratio (OR) with increasing central line usage, ventilator usage, length of stay, and female sex and a negative OR with high income [5].

The overall incidence of HAIs in ICUs has been reduced, but the incidence of *Klebsiella pneumoniae* and *Enterococcus faecium* infection has increased because of the emergence of carbapenem-resistant *K. pneumoniae* (CRKP) and vancomycin-resistant *Enterococcus faecium* (VREfm) in 2019 in Taiwan [6]. Infection control protocols were implemented for the severe acute respiratory syndrome coronavirus (SARS-CoV) pandemic in 2003, and the incidence of HAIs decreased in three medical centers (MCs) in 2004 after the SARS-CoV pandemic [7]. Although 71.9% of coronavirus disease 2019 (COVID-19) patients received antibiotics, 3.4% of COVID-19 patients suffered from bacterial infections [8]. Patients with COVID-19 and HAIs had increased 28-day and in-hospital mortality and longer hospital stays. Steroid use was associated with HAIs, with an OR of 6.97 [9].

The incidence of HAIs decreased after the implementation of an infection prevention and control (IPC) program in Russia and Turkey [10,11]. After the COVID-19 outbreak, the government implemented social distancing, restricted travel, and enhanced healthcare staff protection against HAIs in ICUs in Taiwan. Healthcare providers have compulsively worn N95 masks and protective clothes and have properly engaged in hand hygiene, with alcohol use and washing with soap, during the COVID-19 pandemic. A decline in the incidence of methicillin-resistant *Staphylococcus aureus* (MRSA) was associated with IPC enhancement after the COVID-19 pandemic began [8]. A previous study noted that the incidence of influenza infection decreased during the COVID-19 pandemic and that the mortality owing to influenza and pneumonia decreased [12,13]. A better understanding of the changes in the incidence of HAIs with restrictive policies after the COVID-19 pandemic began could provide a reference for infection control and treatment.

The aim of our study was to analyze data reported by the government to understand the relationship between strict respiration care with more antimicrobial consumption and HAIs. In accordance with the changes in the incidence of DAHAIs, different microorganisms and antimicrobial resistance (AMR) may strengthen infection control in the clinical setting of ICUs.

## 2. Materials and Methods

### Methods

Since 2007, the Taiwanese Nosocomial Infection Surveillance System has been releasing an annual summary of HAIs reported in Taiwan. To address the continuous evolution of medical service content and the expansion of the scope of monitoring, “nosocomial infection” is generally replaced by “healthcare-associated infection” internationally. It was revised in the Taiwan Healthcare-associated Infection and Antimicrobial Resistance Surveillance System by the Taiwanese Center Disease Control in 2020. The surveillance data include those for HAIs reported in the ICUs of 24 MCs and 82 regional hospitals (RHs) since 2021 [14]. The definitions of HAIs followed those of the Taiwan Centre for Disease Control [14]. COVID-19 has been circulating in Taiwan since January 2020. Making use of the open dataset with the annual report, we defined the pre-COVID-19 period as 2015–2019 and the post-COVID-19 period as 2020 to 2022.

**Figure 1:**
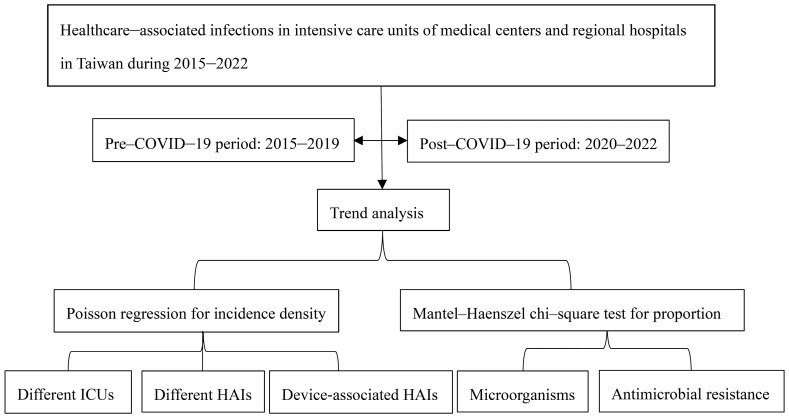
The flowchart of this study.

The incidence density of HAIs, defined as the number of episodes divided by person-days of hospitalization in ICUs, was determined based on numerous different types of infections: bloodstream infections (BSIs), urinary tract infections (UTIs), healthcare-acquired pneumonia (HAP), surgical site infections (SSIs), and infections of other sites in the body [15]. The different types of DAHAIs reported in ICUs are central line-associated BSIs (CLABSIs), catheter-associated UTIs (CAUTIs), and ventilation-associated pneumonia (VAP). The dataset also included the causative pathogens for the HAIs, and different types of AMR observed for the pathogens that cause HAIs in ICUs were also noted for MCs and RHs from 2015 to 2022, which were included in the annual reports from the Taiwan Centre for Disease Control [14]. We considered the Anatomical Therapeutic Chemical codes J01, for all antimicrobial agents, J01C, for beta-lactamase antimicrobial agents, and J01D, for other beta-lactamase antimicrobial agents, during hospitalization for the antimicrobial consumption of Taiwan in 2023 [15]. The annual mean length of hospital stay in ICUs was collected [16]. This study received approval from the Institutional Review Board of Tri-Service General Hospital, under the number TSGH-C202305039. A flowchart is shown in Figure 1.

Poisson regression was performed for changes in the incidence densities of HAIs. The relative risk (RR) was analyzed before and after the COVID-19 pandemic began. The Mantel–Haenszel chi-square test was used to analyze the linear trend in the proportions of microorganisms, and the crude OR was used to explore the risk of percentage change by comparing 2015 and 2022. The correlation between antimicrobial consumption and microorganisms or AMRs was analyzed. Generalized linear regression was performed to determine the associations between HAIs and HAP, BSIs and CLABSIs, and HAP and VAP. Statistical analysis was performed via SPSS version 21 (Asia Analytics Taiwan Ltd., Taipei, Taiwan).

## 3. Results

The overall incidence of HAIs in ICUs ranged from 5.7 to 5.17 episodes/1000 person-days before and after the COVID-19 pandemic (*p* = 0.001). The most common HAIs in 2022 were BSIs, followed by UTIs and HAPs. The incidence of BSIs increased from 2.13/1000 to 2.41/1000 episodes/1000 person-days (*p* < 0.001), and that of HAP decreased from 0.82/1000 to 0.44/1000 episodes/1000 person-days (*p* < 0.001) (Table 1). The incidence densities of HAIs in medical ICUs, surgical ICUs, and cardiac ICUs were higher than the mean incidence density but lower than the average incidence density in general ICUs and pediatric ICUs. The highest number of HAIs occurred in surgical ICUs in 2015, but the highest number of HAIs in 2022 occurred in medical ICUs. The trend in incidence density decreased in surgical, pediatric, and general ICUs, but it increased in medical and cardiac ICUs. (Table 2).

**Figure 2:**
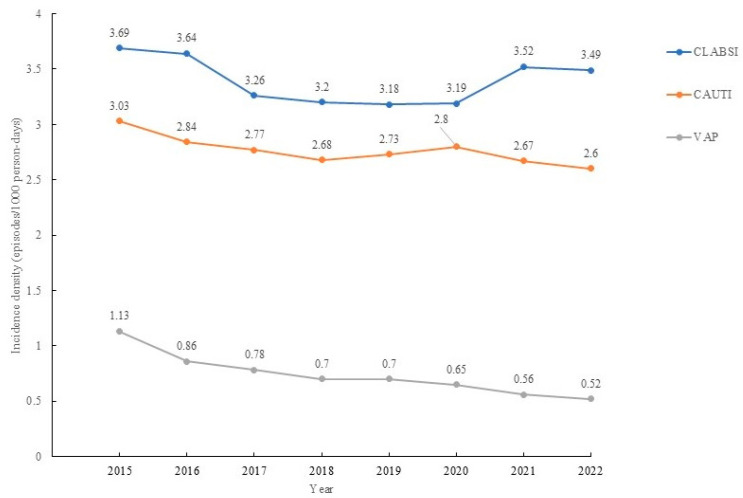
The trends of different types of device-associated healthcare-associated infections.

**Figure 3:**
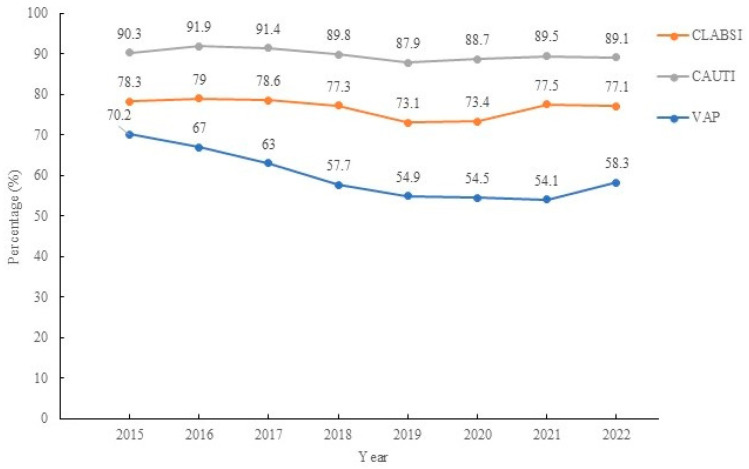
The percentage of device use in different types of device-associated healthcare-associated infections.

The incidence density of CLABSIs decreased from 3.69 episodes/1000 person-days to 3.49 episodes/1000 person-days (*p* = 0.017), and that of VAP (*p* < 0.001) and CAUTIs (*p* = 0.001) decreased. The RR decreased by 0.993 (95% CI: 0.987–0,999, *p* < 0.001) for CLBSIs, decreased by 0.904 (95% CI: 0.893–0.915, *p* < 0.001) for VAP, and decreased by 0.984 (95% CI: 0.979–0.99) from 2015 to 2022 for CAUTI (Figure 2). The usage percentage of devices in device-related BSIs decreased from 78.3% to 77.1% (*p* < 0.001); the percentage of devices used for device-related UTIs decreased from 90.3% to 89.1% (*p* < 0.001), and the percentage of devices used for VAPs decreased from 70.2% to 58.3% (*p* < 0.001) (Figure 3). The overall incidence density of HAIs related to HAP was reduced, with an RR of 1.316 (95% CI: 1.225–1.414, *p* < 0.001). The increase in the incidence of BSIs was related to CLABSIs, with an RR of 1.241 (95% CI: 1.178–1.32, *p* < 0.001), and the decrease in the incidence of HAP was related to VAP, with an RR of 2.324 (95% CI: 2.075–2.602, *p* < 0.001) (Table 3).

**Figure 4:**
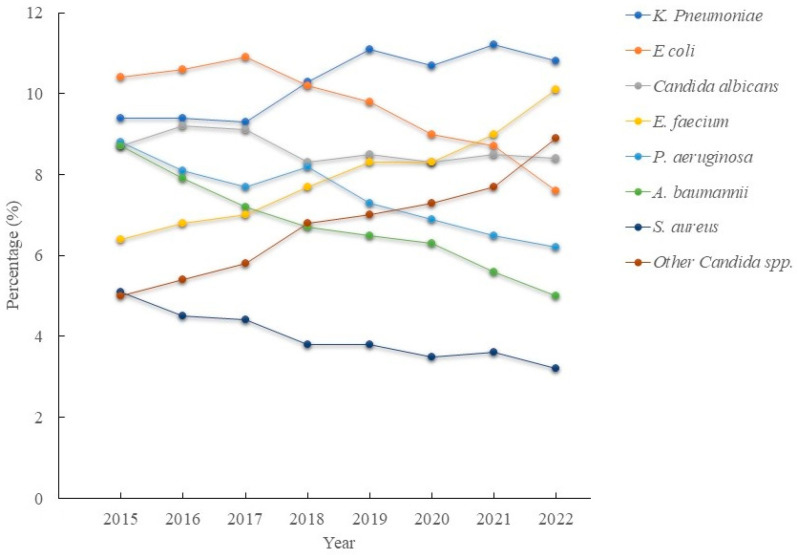
The percentage of different pathogens in healthcare-associated infections in intensive care units from 2015 to 2019.

The top three pathogens in 2022 were *K. pneumoniae*, *Enterococcus faecium*, and other Candida species. The proportion of *E. coli* among the pathogens decreased from 10.4% to 7.6% (*p* < 0.001), with a crude OR of 0.822 (95% CI: 0.75–0.902, *p* < 0.001), while for the proportion during 2022 compared with that during 2015, the proportion of patients with *Pseudomonas aeruginosa* decreased from 8.8% to 6.2% (*p* < 0.001), with a crude OR of 0.68 (95% CI: 0.62–0.762, *p* < 0.001); that of *Staphylococcus aureus* decreased from 5.1% in 2015 to 3.2% in 2022 (*p* < 0.001), with a crude OR of 0.612 (95% CI: 0.533- 0.703, *p* < 0.001); and that of *Acinetobacter baumannii* decreased from 8.7% to 5% (*p* < 0.001), with a crude OR of 0.549 (95% CI: 0.492–0.613, *p* < 0.001), from 2015 to 2022 (Figure 4). The proportion of patients with *K. pneumoniae* increased from 9.4% to 10.8% (*p* < 0.001), with a crude OR of 1.157 (95% CI: 1.059–1.264, *p* = 0.001); that of patients with *E. faecium* increased from 6.4% to 10.1% (*p* < 0.001), with a crude OR of 1.634 (95% CI: 1.48–1.804, *p* < 0.001); and that of patients with other Candida spp. increased from 5% to 8.9% (*p* < 0.001), with a crude OR of 1.858 (95% CI: 1.667–2.071, *p* < 0.001) (Figure 4). AMR analysis revealed that the prevalence of MRSA decreased from 70% to 52.6% (*p* < 0.001), with a crude OR of 0. 475 (95% CI: 0.359–0.629, *p* < 0.001); the prevalence of CRKP increased from 23.2% to 43.7% (*p* < 0.001), with a crude OR of 2.576 (95% CI: 2.14–3.102, *p* < 0.001); the prevalence of VREfm increased from 57.9% to 66.6% (*p* < 0.001), with a crude OR of 1.447 (95% CI: 1.189–1.761, *p* < 0.001); the prevalence of CRAB increased from 71% to 82% (*p* < 0.001), with a crude OR of 1.853 (95% CI: 1.424–2.411, *p* < 0.001); the prevalence of CR *Pseudomonas aeruginosa* increased from 17.8% to 21.3% (*p* = 0.029), with a crude OR of 1.247 (95% CI: 0.972–1.599, *p* = 0.083) and the prevalence of CR *E. coli* increased from 2.1% to 5.9% (*p* < 0.001), with a crude OR of 2.945 (95% CI: 1.786–4.858, *p* < 0.001).

From 2015 to 2022, the defined daily dose (DDD) of all antimicrobial agents increased from 624 to 695 per 1000 person-days, the DDD of β-lactamase antimicrobial agents declined from 164 to 154 per 1000 person-days, and the DDD of other β-lactamase agents increased from 270 to 320.8 per 1000 person-days during hospitalization. Declines in *A. baumannii* and *P. aeruginosa* were negatively associated with antimicrobial use, particularly other β-lactamases, whereas decreased *S. aureus* correlated positively with reduced β-lactamase antimicrobial consumption (Table 4). The correlation between antimicrobial consumption and AMRs showed that CRKP is positively related to all antimicrobial agents and other beta-lactam antimicrobial agents, and MRSA is negatively related to all antimicrobial agents and other beta-lactam antimicrobial agents (Table 5).

A linear regression of HAI incidence and the mean length of hospital stay in Taiwan revealed that every increase of 1 day increased the CRAB incidence by 12.31% (95% CI: 5.01–19.62%, *p* = 0.006), but it did not significantly increase the CRKP incidence by 18.1% (95% CI: −1.057–37.26%, *p* = 0.06), and there was no relationship between VREfm, which increased by 6.17% (95% CI: −10.98–23.32%).

## 4. Discussion

This study revealed that the incidence densities of overall HAIs, HAP, and VAP decreased during the COVID-19 pandemic in Taiwan. The higher quality of infection control in the COVID-19 era was related to the decrease in HAI incidence. The proportions of *E. coli*, *P. aeruginosa*, *S. aures*, and *A. baumannii* decreased, and the percentage of MRSA decreased. However, the incidence densities of BSIs tended to increase, and the percentages of CRKP, CRAB, VREfm, and CR *E. coli* increased. CRKP increased, whereas MRSA decreased in relation to antimicrobial consumption. Understanding the change in the incidence of HAIs in ICUs due to COVID-19 could support a more precise method for empiric antibiotic administration and could lead to the development of aggressive infection control programs.

The incidence of HAIs in China has decreased because COVID-19 patients are admitted to modular hospitals rather than ICUs of general hospitals [17]. In China, HAP accounts for the majority of HAIs, and its incidence decreased during the restriction period [17]. In Taiwan, patients suffering from severe COVID-19 were hospitalized in negative-pressure ICU rooms previously used for respiratory diseases, like tuberculosis, and patients with milder symptoms were isolated at home for 1 to 2 weeks from 2020 to 2022 [18,19]. Restrictive policies resulted in lower disease prevalence from 2020 to 2022, which may have caused the incidence density of overall HAIs to decrease during the COVID-19 pandemic due to strict infection protective policies in Taiwan. The use of suitable sanitizers reduced the incidence of HAP and overall HAIs. Healthcare staff wore sterile clothes and gloves before taking care of patients during the COVID-19 pandemic, reducing contamination of different patients, which may reduce the incidence of seasonal influenza activity [12]. The number of non-COVID-19 HAP patients was reduced in winter, and the patients were older in one regional hospital in southern Taiwan between 2020 and 2019 [12]. Our study revealed a similar decrease in the incidence of HAP. The HAP incidence was reduced in relation to the VAP incidence, and the incidence of overall HAIs was associated with the incidence of HAP in our survey. High-flow nasal cannula (HFNC) use reduced the number of intubations among COVID-19 patients [20]. The frequency of HFNC increased in dyspneic patients during the COVID-19 pandemic, with delay or without ventilators support in COVID-19 patients. HFNC could also alleviate resource constraints and reserve ventilators for the most needy patients [21]. Ventilator use among patients with HAIs showed a significant decline in our study. 

Severe conditions and more comorbidities of patients in medical and surgical ICUs lead to a higher incidence of HAIs than in other ICUs. The trend in HAIs in medical and cardiac ICUs has increased due to the complexity of care for patients with multiple comorbidities, patients with frailty, hemodynamically unstable patients needing central lines for intravenous medications, and patients requiring fluid, nutrient support, central venous pressure monitors, frequent ventilators for airway support, and urinary catheters to record input and output. The higher catheter usage of patients in cardiac ICUs was associated with a higher rate of HAIs. Some patients with COVID-19 initially experienced cardiac symptoms rather than symptoms of COVID-19, increasing the need for isolation by medical staff without the protection of N95 masks at the beginning of the COVID-19 pandemic. The excessive workload required for nurses to provide care for patients in a negative-pressure isolation ward led to a shortage of highly skilled nurses.

During the COVID-19 pandemic, antimicrobial susceptibility among Enterobacterales that cause BSIs in Taiwan showed a general decline [22]. BSIs were not significantly correlated with antimicrobial consumption but were related to CLABSIs in our study. Healthcare providers use central lines to administer intravenous medications and reduce the duration of contact with patients by changing intravenous catheters. In total, 70% of COVID-19 patients received empiric antibiotics [8] and overly relied on second-line antibiotics, which increased the rate of BSIs. The incidence of CLABSIs increased, and that of VAP decreased, in Chang Gang Medical Center during the COVID-19 pandemic [23]. The incidence densities of BSIs related to CLABSIs increased after the COVID-19 pandemic began. It is necessary to reduce central line usage and remove central lines as early as possible in the clinical setting in the post-COVID-19 era, according to this finding.

The previous experience with SARS-CoV in 2003 resulted in an initial decrease followed by an increase in the incidence of *E. coli* and *A. baumannii* infection in three MCs in Taiwan [7]. The incidence of *S. aureus*, *A. baumannii*, and *Pseudomonas aeruginosa* infections decreased before the COVID-19 outbreak in Taiwan [6]. The proportions of *A. baumannii* and *P. aeruginosa* were negatively associated with all and other beta-lactamase antimicrobial consumption. The decreased proportion of *S. aureus* was positively associated with reduced β-lactam use and MRSA decrease (correlation coefficient: 0.884, *p* = 0.004). Antimicrobial use increased the incidence of CR *E. coli* [24]. Our study observed an increasing trend in CR *E. coli* among ICU patients, despite an overall prevalence below 10%, with an OR of 2.9; however, this trend was not significantly associated with antimicrobial consumption. The proportions of *E. coli* decreased, possibly associated with increases in the robustness of IPC programs during the COVID-19 pandemic. The proportions of patients with *E. coli* decreased with respect to BSIs and UTIs. The use of empiric broad-spectrum antimicrobial agents may increase the incidence of HAIs. Ampicillin/sulbactam, imipenem, and levofloxacin usage increased for *A. baumannii*, and tetracycline, erythromycin, and levofloxacin usage increased for *S. pyogenes* in northern Taiwan [25]. The increasing use of teicoplanin and tigecycline was positively related to the incidence of VRE in HAIs [26]. *K. pneumoniae* was the most common microorganism, *E. faecium* was the second most common microorganism, and other Candida species were the third most common microorganisms in the COVID-19 era. The trend of *E. faecium* increased during 2015–2019 [6]; our study showed a similar change after COVID-19, and the incidence of E. faecium was positively associated with antimicrobial consumption and VREfm (correlation coefficient: 0.747, *p* = 0.033). The proportion of *K. pneumoniae* increased from third to first. The incidence of *K. pneumoniae* was associated with lower antimicrobial susceptibility in a previous study [27]. Our study showed a similar finding related to CRKP, with a correlation coefficient of 0.932, *p* = 0.001. The incidence of infection with other Candida species increased from the top seven to the top three of overall HAIs, indicating an increase in the incidence of opportunistic infections after immune suppression agents were used. The steroids for anti-inflammation protection used frequently in patients with COVID-19 seem to cause a greater percentage of fungal infections. The use of antifungal agents increased, as reflected in the national antimycotic data for Taiwan [15]. Patients with Candidemia had higher rates of death related to disease severity and underlying comorbidities [28].

MRSA decreased in abundance at Chang Gang Medical Center and Taiwan University Hospital after the SARS-CoV outbreak [23,29]. The increased consumption of teicoplanin and linezolid was negatively associated with the incidence of MRSA in patients with HAIs [26]. The incidence of MRSA decreased in relation to the antimicrobial consumption in our study. It is also potentially related to alcohol sanitizer use and hand washing among healthcare workers [8].

In 2005, the proportion of *Enterobacteriaceae* producing extended-spectrum beta-lactamase (ESBL) was 26% among *K. pneumoniae* in Taiwan [30]. CRAB, VRE, CRKP, and CR *E. coli* increased in central Taiwan [31]. Our study confirmed this observation. The incidence of ESBL-producing *K. pneumoniae* increased during the COVID-19 pandemic due to the use of antibiotics in seventy percent of patients [8]. Antimicrobial use increased CRKP incidence [25]; our study showed a similar finding. Moreover, antibiotic usage has increased specifically in Taiwan [15], possibly increasing the incidence of CRKP. COVID-19 patients have been admitted to negative-pressure isolation ICUs with empiric antimicrobial therapy in MCs, and CRKP incidence in one MC with a more robust IPC program showed no significant change [32]. ICU admission, the use of multiple antimicrobial agents, and the use of devices are related to CRKP risk [33]. The susceptibility of *K*. *pneumoniae* detected in MCs (72%) to amoxicillin/clavulanate is greater than that detected in RHs (49%) and LHs (55%) [34]. Our study revealed a 2.6-fold increase in the risk of CRKP infection in ICUs. Chlorhexidine gluconate (CHG) use can reduce the incidence of KPC-producing enterobacterial HAIs from 5.01 to 2.25 episodes/1000 person-days, but there is no difference in the incidence of device-induced HAIs [35]. CHG baths seem to be a way to reduce CRKP incidence. They demonstrated an upward trend in the incidence of New Delhi metallo-β-lactamase-producing carbapenem-resistant Enterobacterales and CRAB during the COVID-19 pandemic [8]. Higher bed turnover and higher bed occupancy were associated with a lower incidence of HAIs in Iran [36]. Our study showed that CRAB was not associated with antimicrobial consumption but was correlated with the length of hospital stay. Healthcare providers need to treat patients with effective medication and procedures to reduce the length of stay and increase the bed turnover rate to promote lower HAI incidence. Improving the surveillance of AMR in ICUs is essential because the application of suitable antimicrobial therapy could reduce the length of stay in ICUs.

There were several limitations in our study. First, we surveyed an open dataset with the absence of individual-level and outcome data, which did not provide the patients’ ages, comorbidities, disease severity, surgical complexity, or device days; thus, these risk factors were not analyzed. It may be over-interpreted by average data. Second, data from the ICUs of MCs and RHs, data from LHs with lower reporting rates, and data from general wards were unavailable. However, more than 80% of all ICUs in MCs and RHs account for the majority of ICUs [6], and there were 2.6-fold more HAIs in ICUs than in general wards (1.99/1000 person-days) in 2021 [14]. Third, the relationships between HAIs and HAP, HAP and VAP, or BSIs and CLABSIs are associative rather than cause–effect relationships, and further studies are needed to confirm the nature of these relationships. Fourth, the prevalence of CRKP increased in relation to that of empiric antimicrobial agent use, and this possible cause–effect association needs to be confirmed in further studies. Fifth, owing to the use of the open dataset with an annual HAI report, we were unable to obtain and explore the cumulative monthly data before and after the intervention following COVID-19. The monthly data obtained by the Taiwan CDC showed that CRKP increased and MRSA decreased during COVID-19 [37].

## 5. Conclusions

The incidence of overall HAIs and HAP showed a downward trend during the period of the COVID-19 pandemic with strict ICPs in Taiwan. It is essential for infection control to support HAI surveillance in ICUs. Strict care of respiratory disorders related to a lower rate of HAIs in ICUs, following general care for COVID-19, and the prevention of HAIs has become important. Continuous monitoring and the implementation of effective ICPs, vaccination, and stewardship interventions regarding antibiotic usage reduce unnecessary device use. According to recent findings, these measures will help to decrease the incidence of HAIs, and AMRs in unrestricted periods.

## Figures and Tables

**Table 1:** The incidence densities of healthcare-associated infections before and after coronavirus disease 2019.

	2015	2016	2017	2018	2019	2020	2021	2022	*p*
All HAIs	5.7	5.29	4.93	5.19	5.41	5.2	5.26	5.17	<0.001 *
BSIs	2.13	2.11	1.93	1.99	2.11	2.17	2.83	2.41	<0.001 *
UTIs	2.08	1.92	1.84	1.87	1.92	1.95	2.05	1.87	0.222
HAPs	0.82	0.65	0.59	0.61	0.61	0.56	0.53	0.44	<0.001 *
SSIs	0.26	0.26	0.24	0.26	0.25	0.39	0.43	0.23	<0.001 *

* *p* < 0.05. HAIs: healthcare-associated infections; BSIs: bloodstream infections; UTIs: urinary tract infections; HAPs: hospital-acquired infections; SSIs: surgical skin infections.

**Table 2:** The incidence density of healthcare-associated infections in different intensive care units during 2015–2022.

	2015	2016	2017	2018	2019	2020	2021	2022	*p*
Medical ICUs	6.12	5.52	5.44	5.44	5.94	5.92	6.29	6.12	<0.001 *
Surgical ICUs	6.94	6.49	5.93	6.68	6.51	6.26	6.21	6	<0.001 *
Cardiac ICUs	5.07	5.05	4.55	5.23	5.24	6.08	6.5	5.87	<0.001 *
Pediatric ICUs	2.61	2.35	2.16	2.08	2.09	1.96	2.21	1.97	<0.001 *
General ICUs	5.64	5.36	4.88	4.89	5.51	4.94	4.62	4.84	<0.001 *
Overall	5.7	5.29	4.93	5.19	5.41	5.2	5.26	5.17	<0.001 *

* *p* < 0.05. Incidence density: episodes/1000 person-days; ICUs: intensive care units.

**Table 3:** The relationship between different healthcare-associated infections.

	Relative Risk	*p*
Healthcare associated infections related to hospital-acquired pneumonias	1.316 (95% CI: 1.225–1.414)	<0.001 *
Bloodstream infections related to central line associated bloodstream infections	1.241 (95% CI: 1.178–1.32)	<0.001 *
Hospital acquired pneumonias related to ventilator-associated pneumonias	2.324 (95% CI: 2.075–2.602)	<0.001 *

* *p* < 0.05.

**Table 4:** The correlation between antimicrobial consumption and bloodstream infections or microorganisms.

ATC code	BSIs	*K. pneumoniae*	*E. faecium*	*E. coli*	*A. baumannii*	*P. aeruginosa*	*S. aureus*
J01	0.679	0.795	0.986	−0.948	−0.981	−0.995	−0.925
*p*	0.321	0.205	0.014 *	0.052	0.019 *	0.005 *	0.075
J01C	−0.66	−0.947	−0.925	0.798	0.974	0.973	0.958
*p*	0.34	0.053	0.075	0.202	0.026 *	0.027 *	0.042 *
J01D	0.704	0.791	0.979	−0.946	−0.976	−0.992	−0.914
*p*	0.321	0.209	0.021 *	0.054	0.024 *	0.008 *	0.086

* *p* < 0.05; ATC code: Anatomical Therapeutic Chemical code; BSIs: bloodstream infections.

**Table 5:** The correlation between antimicrobial consumption and antimicrobial-resistant microorganisms.

ATC code	CRKP	MRSA	VREfm	CRAB	CRPA	CR *E. coli*
J01	0.97	−0.992	0.746	0.879	0.83	0.895
*p*	0.03 *	0.008 *	0.254	0.121	0.17	0.105
J01C	−0.98	0.945	−0.919	−0.722	−0.937	−0.768
*p*	0.02 *	0.055	0.081	0.278	0.063	0.232
J01D	0.964	−0.995	0.742	0.867	0.818	0.908
*p*	0.036 *	0.005 *	0.258	0.133	0.182	0.092

* *p* < 0.05; ATC code: Anatomical Therapeutic Chemical code; CRKP: carbapenem-resistant *K. pneumoniae*; MRSA: methicillin-resistant *Staphylococcus aureus*; VREfm: vancomycin-resistant *Enterococcus faecium*; CRAB: carbapenem-resistant *Acinetobacter baumannii*; CRPA: carbapenem-resistant *Pseudomonas aeruginosa*; CR *E. coli*: carbapenem-resistant *Escherichia coli*.

## Data Availability

The healthcare-associated data from intensive care units in Taiwan, 2022, can be obtained at https://www.cdc.gov.tw/En/Category/Page/J63NmsvevBg2u3I2qYBenw (accessed on 1 April 2023).

## Abbreviations

The following abbreviations are used in this manuscript:

HAIs	Healthcare-associated infections
DAHAIs	Device-associated HAIs
ICU	Intensive care unit
OR	Odds ratio
CRKP	Carbapenem-resistant *K. pneumoniae*
VREfm	Vancomycin-resistant *Enterococcus faecium*
SARS-CoV	Severe acute respiratory syndrome coronavirus
MCs	Medical centers
COVID-19	Coronavirus disease 2019
MRSA	Methicillin-resistant *Staphylococcus aureus*
AMR	Antimicrobial resistance
RHs	Regional hospitals
BSIs	Bloodstream infections
UTIs	Urinary traction infections
HAP	Healthcare-acquired pneumonia
CLABSIs	Central line-associated BSIs
CAUTIs	Catheter-associated UTIs
VAPs	Ventilation-associated pneumonia
CRAB	Carbapenem-resistant *Acinetobacter baumannii*
RR	Relative risk
HFNC	High-flow nasal cannula
DDD	Defined daily dose
ESBL	Extended-spectrum beta-lactamase
CHG	Chlorhexidine gluconate

## References

[1] Haque M., McKimm J., Sartelli M., Dhingra S., Labricciosa F.M., Islam S., Jahan D., Nusrat T., Chowdhury T.S., Coccolini F. (2020). Strategies to prevent healthcare-associated infections: A narrative overview. Risk Manag. Heal. Policy.

[2] Liu X., Shrestha R., Koju P., Maharjan B., Shah P., Thapa P., Li H. (2022). The direct medical economic burden of healthcare-associated infections and antimicrobial resistance: A preliminary study in a teaching hospital of Nepal. J. Glob. Antimicrob. Resist..

[3] Wang Y.-C., Shih S.-M., Chen Y.-T., Hsiung C.A., Kuo S.-C. (2020). Clinical and economic impact of intensive care unit-acquired bloodstream infections in Taiwan: A nationwide population-based retrospective cohort study. BMJ Open.

[4] MacEwan S.R., Beal E.W., Gaughan A.A., Sieck C., McAlearney A.S. (2022). Perspectives of hospital leaders and staff on patient education for the prevention of healthcare-associated infections. Infect. Control Hosp. Epidemiol..

[5] Rosenthal V.D., Jin Z., Memish Z.A., Daboor M.A., Al-Ruzzieh M.A., Hussien N.H., Guclu E., Olmez-Gazioglu E., Ogutlu A., Agha H.M. (2022). Risk factors for mortality in ICU patients in 10 middle eastern countries: The role of healthcare-associated infections. J. Crit. Care.

[6] Lin Y.-R., Lin Y.-Y., Yu C.-P., Yang Y.-S., Cheng C.-G., Cheng C.-A. (2021). In Increased Involvement of Klebsiellapneumoniae and Enterococcusfaecium in Healthcare-Associated Infections of Intensive Care Units in Taiwan. Healthcare.

[7] Lai C., Chen Y., Lin S., Chung K., Sheng W., Ko W., Hsueh P.R. (2014). Changing aetiology of healthcare-associated bloodstream infections at three medical centres in Taiwan, 2000–2011. Epidemiol. Infect..

[8] O’Toole R.F. (2021). The interface between COVID-19 and bacterial healthcare-associated infections. Clin. Microbiol. Infect..

[9] Huang R.-C., Chiu C.-H., Chiang T.-T., Tsai C.-C., Wang Y.-C., Chang F.-Y., Yang Y.S., Wang C.H. (2022). Hospital-acquired infections in patients hospitalized with COVID-19: First report from Taiwan. J. Chin. Med. Assoc..

[10] Ershova K., Savin I., Kurdyumova N., Wong D., Danilov G., Shifrin M., Alexandrova I., Sokolova E., Fursova N., Zelman V. (2018). Implementing an infection control and prevention program decreases the incidence of healthcare-associated infections and antibiotic resistance in a Russian neuro-ICU. Antimicrob. Resist. Infect. Control..

[11] Gozel M.G., Hekimoglu C.H., Gozel E.Y., Batir E., McLaws M.L., Mese E.A. (2021). National Infection Control Program in Turkey: The healthcare associated infection rate experiences over 10 years. Am. J. Infect. Control..

[12] Huang C. (2021). The COVID-19 pandemic and the incidence of the non-COVID-19 pneumonia in adults. Front. Med..

[13] Gao W., Sanna M., Huang G., Hefler M., Tsai M.-K., Wen C.-P. (2021). Examining population health during the COVID-19 pandemic: All-cause, pneumonia and influenza, and road traffic deaths in Taiwan. Ann. Intern. Med..

[14] Taiwan Centers of Disease Control (2022). The Healthcare-Associated of Intensive Care Units in Taiwan. https://www.cdc.gov.tw/En/Category/Page/J63NmsvevBg2u3I2qYBenw.

[15] Taiwan Centers of Disease Control (2023). The Antimicrobial Consumptions of Taiwan in 2023. https://www.cdc.gov.tw/.

[16] Taiwan Ministry of Health and Welfare Survey on Service Volume of Medical Institutions in 2022. https://www.gender.ey.gov.tw/gecdb/Stat_Statistics_Category.aspx?fs=fTQP3HmkUvd1PbnmtSP3rw%40%40&cs1=aPi33EfnEATKPKjm9jJFBA%40%40&cs2=FuWFCi3De1SSQR5qlMyr0g%40%40).

[17] Rong R., Lin L., Yang Y., Zhao S., Guo R., Ye J., Zhu X., Wen Q., Liu D. (2023). Trending prevalence of healthcare-associated infections in a tertiary hospital in China during the COVID-19 pandemic. BMC Infect. Dis..

[18] Tsou H.H., Kuo S.C., Lin Y.H., Hsiung C.A., Chiou H.Y., Chen W.J., Wu S.I., Sytwu H.K., Chen P.C., Wu M.H. (2022). A comprehensive evaluation of COVID-19 policies and outcomes in 50 countries and territories. Sci. Rep..

[19] Taiwan Infectious Disease Thematic Database Isolation Policies in Taiwan. https://tidtd.nhri.edu.tw/quarantine-policy-timeline-2/.

[20] Li Y., Li C., Chang W., Liu L. (2023). High-flow nasal cannula reduces intubation rate in patients with COVID-19 with acute respiratory failure: A meta-analysis and systematic review. BMJ Open.

[21] Liu C.W., Cheng S.L. (2022). Application of High-Flow Nasal Cannula in COVID-19: A Narrative Review. Life.

[22] Lee Y.L., Liu C.E., Tang H.J., Huang Y.T., Chen Y.S., Hsueh P.R., SMART Taiwan Group (2024). Epidemiology and antimicrobial susceptibility profiles of Enterobacterales causing bloodstream infections before and during COVID-19 pandemic: Results of the Study for Monitoring Antimicrobial Resistance Trends (SMART) in Taiwan, 2018–2021. J. Microbiol. Immunol. Infect..

[23] Chen Y.-Y., Chen L.-Y., Lin S.-Y., Chou P., Liao S.-Y., Wang F.-D. (2012). Surveillance on secular trends of incidence and mortality for device–associated infection in the intensive care unit setting at a tertiary medical center in Taiwan, 2000–2008: A retrospective observational study. BMC Infect Dis..

[24] Lai C.-C., Chen S.-Y., Ko W.-C., Hsueh P.-R. (2021). Increased antimicrobial resistance during the COVID-19 pandemic. Int. J. Antimicrob. Agents.

[25] Taiwan Centers of Disease Control (2022). Antimicrobial Resistance Surveillance Annual Report, Taiwan in 2021–2022. https://www.cdc.gov.tw/Category/MPage/4G8HuDdUN1k4xaBJhbPzKQ.

[26] Lee M.C., Chang H., Sun F.J., Wu A.Y., Lu C.H., Lee C.M. (2022). Association between Antimicrobial Consumption and the Prevalence of Nosocomial Carbapenem-Resistant *Escherichia coli* and *Klebsiella pneumoniae* in a Tertiary Hospital in Northern Taiwan. Am. J. Trop. Med. Hyg..

[27] Lai C.-C., Chu C.-C., Cheng A., Huang Y.-T., Hsueh P.-R. (2015). Correlation between antimicrobial consumption and incidence of health-care-associated infections due to methicillin-resistant *Staphylococcus aureus* and vancomycin-resistant enterococci at a university hospital in Taiwan from 2000 to 2010. J. Microbiol. Immunol. Infect..

[28] Hii M., Chang H.-L., Lin L.-C., Lee Y.-L., Liu Y.-M., Liu C.-E., Chen C.H., Cheng Y.R., Chang C.Y. (2015). Changing epidemiology of candidemia in a medical center in middle Taiwan. J. Microbiol. Immunol. Infect..

[29] Chen C.-J., Yang Lauderdale T.-L., Huang Y.-C. (2021). Evolution and population structures of prevalent methicillin-resistant *Staphylococcus aureus* in Taiwan. Front. Microbiol..

[30] Jean S.-S., Hsueh P.-R., Lee W.-S., Chang H.-T., Chou M.-Y., Chen I.-S., Wang J.H., Lin C.F., Shyr J.M., Ko W.C. (2009). Nationwide surveillance of antimicrobial resistance among Enterobacteriaceae in intensive care units in Taiwan. Eur. J. Clin. Microbiol. Infect. Dis..

[31] Tseng Y.W., Huang C.W., Chen C.C., Er T.K. (2024). Assessment of antibiotic resistance patterns in Central Taiwan during the COVID-19 pandemic: A retrospective study. J. Infect. Public Health.

[32] Chang H.C., Chang C.H., Tien K.L., Tai C.H., Lin L.M., Lee T.F., Ku S.C., Fang C.T., Chen Y.C., Sheng W.H. (2024). Impact of coronavirus disease 2019 (COVID-19) on antimicrobial resistance among major pathogens causing healthcare-associated infection. J. Formos. Med. Assoc..

[33] Zhu W.-M., Yuan Z., Zhou H.-Y. (2020). Risk factors for carbapenem-resistant *Klebsiella pneumoniae* infection relative to two types of control patients: A systematic review and meta-analysis. Antimicrob. Resist. Infect. Control.

[34] Taiwan Centers of Disease Control (2022). Antimicrobial Agents Usage in Taiwan During 2021. https://www.cdc.gov.tw/File/Get?q=t9WnCInvvVMS9kUboNEwG4WgpE7_jCv77LnPRq-ahA8qJsp16pQaURPNoq-YXOk8rOPxRYaU05iElAmaD7nRv1JU1ssScWXEfG6JYcOdavjeKlsgjBb32PCAIM_hzeWQnzxC76d15dplS75hosZOMQ.

[35] Reis M.A.O., de Almeida M.C.S., Escudero D., Medeiros E.A. (2022). Chlorhexidine gluconate bathing of adult patients in intensive care units in São Paulo, Brazil: Impact on the incidence of healthcare-associated infection. Braz. J. Infect. Dis..

[36] Izadi N., Eshrati B., Mehrabi Y., Etemad K., Hashemi-Nazari S.S. (2021). The national rate of intensive care units-acquired infections, one-year retrospective study in Iran. BMC Public Health.

[37] Taiwan Centers of Disease Control (2022). The Antimicrobial Resistant of Taiwan During COVID-19. https://www.cdc.gov.tw/File/Get?q=t9WnCInvvVMS9kUboNEwG6kcWFLUQ2PuJpCrSHkBMhj77SNvH3TjC1xNyquh63r8npxSKk3K4tbIn3Cw-FyuUev53IhkKNHrO1LvG7esSWGKc7ZVNdeH95zVA1tWv2Zw_naR6r8NwtjXEozg6NH2VdxBBEAXzykEpFfv4S4X-VNznSjKYjkotxRwk43i6jhWJ_BEZ-DOxtkM824nT03-_Q.

